# Cherubism as a systemic skeletal disease: evidence from an aggressive case

**DOI:** 10.1186/s12891-020-03580-z

**Published:** 2020-08-21

**Authors:** Anne Morice, Aline Joly, Manon Ricquebourg, Gérard Maruani, Emmanuel Durand, Louise Galmiche, Jeanne Amiel, Yoann Vial, Hélène Cavé, Kahina Belhous, Marie Piketty, Martine Cohen-Solal, Ariane Berdal, Corinne Collet, Arnaud Picard, Amelie E. Coudert, Natacha Kadlub

**Affiliations:** 1 Laboratoire de Physiopathologie Orale Moléculaire, INSERM UMRS 1138, Equipe 5, Centre de Recherche de Cordeliers, 75006 Paris, France; 2 Université Paris Descartes, 75006 Paris, France; 3 APHP, Necker Enfants Malades, Service de Chirurgie Maxillo-faciale et Plastique, 75015 Paris, France; 4 APHP, CRMR des Malformations Rares de la Face et de la Cavité Buccale, 75015 Paris, France; 5 BIOSCAR, INSERM U1132, Université de Paris, Hôpital Lariboisière, 75010 Paris, France; 6 Service de Biochimie et Biologie Moléculaire, CHU-Paris-GH Saint Louis Lariboisière Widal, Paris, France; 7 Institut Necker Enfants-Malades, INSERM U1151 – CNRS UMR 8253, Université Paris Descartes-Sorbonne Paris Cité, 75014 Paris, France; 8 Service de Physiologie, Hôpital Necker - Enfants Malades and Hôpital Européen Georges Pompidou, Assistance Publique-Hôpitaux de Paris, 75015 Paris, France; 9 IR4M – Université Paris-Sud, CNRS, Université Paris-Saclay, F91401 Orsay, France; 10 APHP, Necker Enfants Malades, Service d’Anatomopathologie et cytologie, 75015 Paris, France; 11 APHP, Necker Enfants Malades, Département de Génétique Médicale, 75015 Paris, France; 12 APHP, Hôpital Robert Debré, Département de Génétique, 75019 Paris, France; 13 INSERM UMR 1131, Institut de Recherche Saint-Louis, Université de Paris, Paris, France; 14 APHP, Necker Enfants Malades, Service d’imagerie médicale pédiatrique, 75015 Paris, France; 15 APHP, Necker Enfants Malades, Service des Explorations Fonctionnelles, 75015, Paris, France; 16 UFR Odontologie, Garancière, Université Paris Diderot, 75006 Paris, France

**Keywords:** Cherubism, SH3BP2 protein, Systemic inflammation, Bone loss phenotype, Case report

## Abstract

**Background:**

Cherubism is a rare autosomal dominant genetic condition caused by mutations in the *SH3BP2* gene. This disease is characterized by osteolysis of the jaws, with the bone replaced by soft tissue rich in fibroblasts and multinuclear giant cells. SH3BP2 is a ubiquitous adaptor protein yet the consequences of SH3BP2 mutation have so far been described as impacting only face. Cherubism mouse models have been generated and unlike human patients, the knock-in mice exhibit systemic bone loss together with a systemic inflammation.

**Case presentation:**

In light of these observations, we decided to search for a systemic cherubism phenotype in a 6-year-old girl with an aggressive cherubism. We report here the first case of cherubism with systemic manifestations. Bone densitometry showed low overall bone density (total body Z-score = − 4.6 SD). Several markers of bone remodelling (CTx, BALP, P1NP) as well as inflammation (TNFα and IL-1) were elevated. A causative second-site mutation in other genes known to influence bone density was ruled out by sequencing a panel of such genes.

**Conclusions:**

If this systemic skeletal cherubism phenotype should be confirmed, it would simplify the treatment of severe cherubism patients and allay reservations about applying a systemic treatment such as those recently published (tacrolimus or imatinib) to a disease heretofore believed to be localised to the jaws.

## Background

Cherubism (OMIM #118400) is a rare autosomal dominant genetic condition caused by mutations in the *SH3BP2* gene, encoding for an adaptator protein, SH3BP2 [1]. Progressive painless bilateral enlargement of the jawbones characterizes the disease. During the growing phase, the jawbone is replaced by a granuloma containing multinucleated giant cells within a fibrous stroma [2]. Cherubism is described as a maxillofacial localized disease, only affecting jaw bones, and follows a natural course of expansion (from 2 years old to puberty), stabilization and regression. However, its penetrance and expressivity are widely variable from non-symptomatic to lethal cases [3, 4]. In addition to the maxillofacial involvement, general manifestations may occur, especially respiratory disorders mostly due to obstructive apnea [5].

Recent studies carried out on mouse models, have uncovered crucial molecular and cellular aspects of the pathogenic mechanism of cherubism, highlighting auto-inflammatory aspects of the disease and the bone involvement [6]. Notably, cherubism mice exhibit global bone loss and macrophage infiltration of internal organs [7]. However, these observations have never been reported in human cherubism, although interestingly, some authors had partially explored the bone and inflammatory markers, in order to exclude differential diagnosis, such as parathyroid impairment [2, 8–11]. From these studies, we know that standard blood count, serum electrolytes, serum calcium and phosphate concentrations, and TSH, FSH, LH, PTH, PTHrP, T4 and T3 hormones, calcitonin and osteocalcin levels are all within the normal range [8–10], and that alkaline phosphatase is increased [2, 9–11]. However, systemic exploration of cherubism patient is extremely rare and incomplete. Since cherubism is caused by mutations of a ubiquitous adaptator protein involved in bone remodelling and inflammation and the knock-in mice show a systemic bone loss, we investigated potential systemic manifestations of cherubism in a young patient. Here we report a case of aggressive cherubism case associated with systemic inflammatory and bone loss features.

## Case presentation

## Methods

### Phenotype and evaluation of the disease extension

Assessment of the cherubism phenotype was based on clinical and paraclinical data collected retrospectively on a 6 year-old Gabonese girl admitted to our referral center (Hôpital Necker – Enfants Malades) with rapid and expansive swelling affecting the upper and lower jaws bilaterally. A standard CT scan was performed to determine the extension of the disease. To evaluate functional consequences, polysomnography and ophthalmologic examinations were performed. Histopathological data and a genetic testing confirmed the cherubism diagnosis.

### Genetic testing: sequencing analysis

DNA samples were obtained from peripheral leucocytes. Mutation screening was performed by direct bidirectional sequencing of exons and their flanking intron–exon boundaries. The entire coding region of SH3BP2 was examined. Primers sequences and PCR conditions are available on request.

The PCR products were sequenced (Big Dye Terminator Cycle Sequencing Ready Reaction Kit (Applied Biosystems, Foster City, CA, USA), and the reaction products were run on an automated capillary sequencer (ABI 3100 Genetic Analyzer, Applied Biosystems). Sequences were aligned using Seqscape analysis software (Applied Biosystems) and compared with the reference sequences for genomic DNA. The GenBank accession number for the genomic and mRNA sequence reference is SH3BP2 NM_003023.4.

### Histopathological analysis

Formalin-fixed paraffin-embedded samples from the surgical curettage were used for pathological analyses. Sections 4 μm in thickness were stained with hematoxylin/eosin/saffron (HES). Immunohistochemical analyses were conducted with a Leica Bond automated immunohistochemistry slide-processing platform (Bond Max, Leica, Germany) according to the manufacturer’s instructions. Counterstaining was performed using haematoxylin. Sections were incubated with anti-NFATc1 antibody (clone 7A6, 1/100, Santa Cruz, CA, USA) and visualized using a diaminobenzidine tetrahydrochloride chromogen (DAB, SK-4105 Vector Laboratories, Burlingame, CA, USA).

### Biological investigations

Standard biochemical investigations (blood formula and serum electrolytes as well as urine testing) were performed according conventional methods and adjusted to age.

Inflammation, bone remodelling and mineral metabolism studies were performed in the same center. All results were adjusted to age, except 25-hydroxyvitamin D and PTH (age-independent variations).

### Radiological investigations

Abdominal ultrasound-scans were performed to detect hepatosplenomegaly. Areal bone mineral density (aBMD) was assessed using dual-energy X-ray absorptiometry (DXA) with a Lunar Prodigy Advance DXA system (GE Lunar Corp., Madison, WI, USA); aBMD Z-scores were adjusted for height, age and black ethnic origin (Lunar, female pediatric reference curve adjusted for black ethnic origin).

### Exclusion of differential diagnosis

Differential diagnosis of the jawbone lesions was excluded by histopathological examination.

Infectious diseases were excluded by adapted explorations that were oriented by the geographic origin of the patient.

### Next generation sequencing gene panel

An NGS-based high-throughput custom bone mass panel containing 50 genes known or suggested to cause alteration in bone mineral density was interrogated. The genes included in the panel are *ALPL, AMER1, ANKH, BMP1, CA2, CLCN7, COL1A1, COL1A2, CRTAP, CTSK, CYP27B1, DKK1, DMP1, EN1, FAM20C, FGF23, FKBP10, GJA1, HPGD, IFITM5, LEMD3, LRP4, LRP5, LRP6, OSTM1, P3H1, PHEX, PLEKHM1, PPIB, RUNX2, SERPINF1, SFRP4, SLC34A1, SLCO2A1, SNX10, SOST, SP7, SQSTM1, TCIRG1, TGFB1, TNFRSF11A, TNFRSF11B, TNFSF11, VDR, WLS, WNT1, WNT11, WNT16, WNT3A, XYLT2.* This gene panel is adapted from [12].

## Results

### Cherubism diagnosis

#### Phenotype and evaluation of disease extension

A 6 year-old Gabonese girl presented with rapid and progressive swelling of the jaw, evolving since the age of 3. Her height was 109 cm (− 1.7 SD) and weight was 15.2 kg (− 2 SD). Her weight curve was normal from birth to the age of 3, when she began to present cherubism lesions. Further inflection of height and weight curves was observed at the age of 6, when cherubism lesions became more enlarged and aggressive. No clinical signs of undernourishment were observed.

The patient presented with a family history of cherubism: her mother, grandmother and uncle presented mild forms of cherubism, while her cousin, managed in another center, presented with an aggressive form of cherubism without skeletal or inflammatory anomalies. The other family members were unaffected by short stature or skeletal disease.

**Fig. 1:**
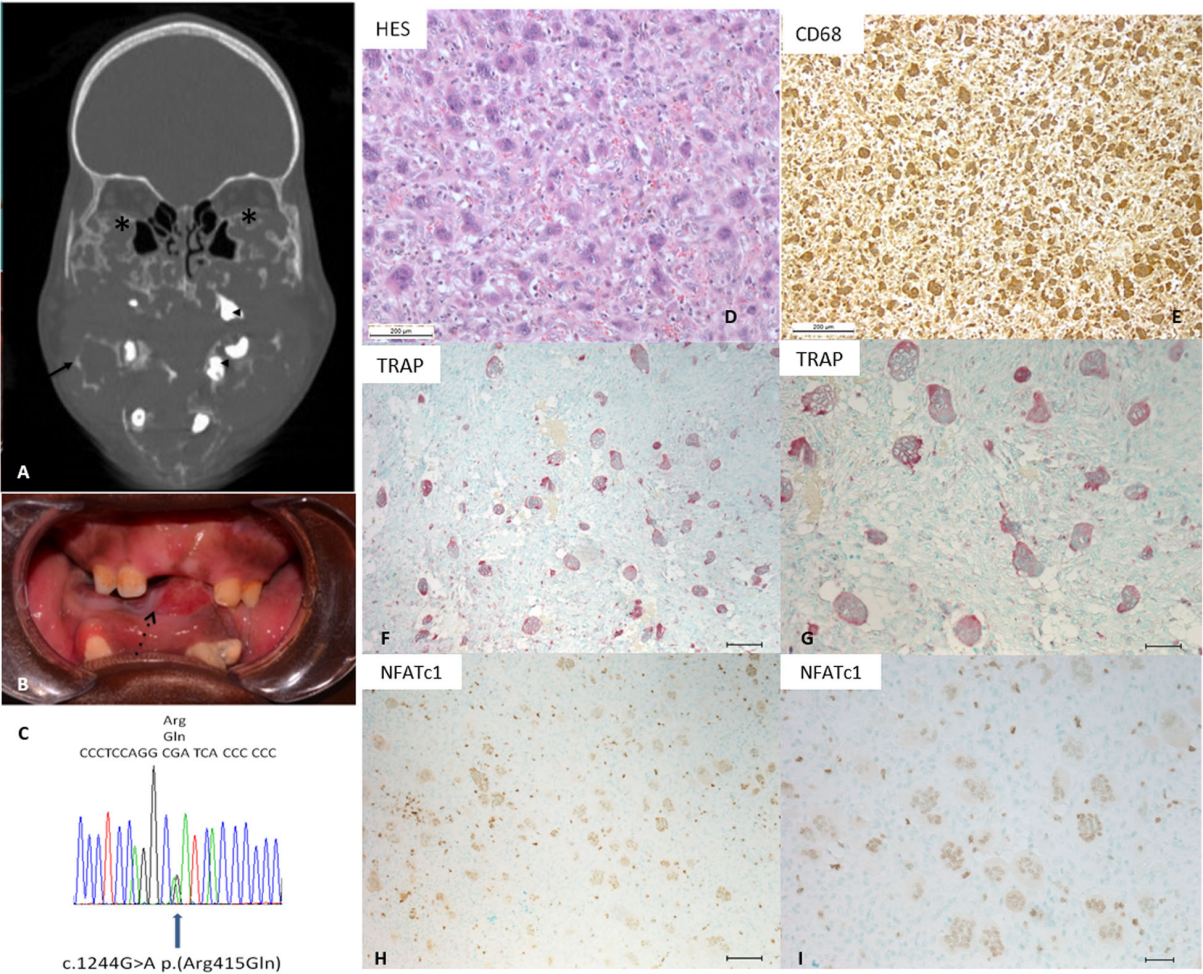
Classic features of severe cherubism. **a** and **b** Clinical and radiological cherubism features. Coronal preoperative CT scan (**a**) showing multilocular tissue expansion developing into the maxilla and the mandible, with osteolysis and cortical bone expansion (arrows), tooth displacements (arrow heads), and elevation of the orbital floors (*). Intraoral examination (**b**), showing symmetrical enlargement of the maxilla and mandible causing proptosis and glossoptosis due to expansion through the floor of the mouth (dotted arrow). **c** Electropherogram showing the recurrent pathogenic mutation c.1244 p.(Arg415Gln) in the *SH3BP2* gene. Identification of this previously described point mutation confirmed the cherubism diagnosis. **d**-**i** Histological cherubism features. **d** Cell-rich areas are composed of oval to spindle-shaped fibroblasts and numerous osteoclast-like giant multinucleated cells . Numerous vessels are noted, sometimes surrounded by perivascular hyalinosis (HE Staining, scale bar 200 μm). **e** CD68 positive cells revealed by immunochemistry**.** Most of the giant multinucleated cells are CD68 positive. **f** and **g** TRAP staining. The giant multinucleated cells are TRAP positive. **f** Low magnification, scale bar 100 μm. **g** High magnification, scale bar 50 μm (counterstaining with methyl green). **h** and **i** Immunohistochemistry with anti-NFATc1 antibody. A nuclear staining is observed in most of the giant multinucleated cells. **h** low magnification, scale bar 100 μm. **i** high magnification, scale bar 50 μm (counterstaining with methyl green)

Clinical examination revealed symmetrical and painless enlargement of the maxilla, causing complete bilateral nasal obstruction, proptosis and enlargement of the mandible causing glossoptosis (Fig. 1). The patient was not pyretic and presented no arthritis. Polysomnography was subnormal with only one obstructive apnea per hour. Ophthalmologic examination revealed exophthalmos with a scleral-show and an impairment of ocular mobility in both eyes. Dilated fundus examination was normal. The patient presented decreased visual acuity (3/10 in both eyes).

A craniofacial CT-scan revealed multilocular tissue expansion developing in the maxilla and the mandible, responsible for both osteolysis and cortical bone thinning and expansion, tooth displacements and tooth root resorption. The lesion expanded through the floor of the mouth and into the nose, leading to upper airway obstruction and elevation of the orbital floor (Fig. 1). Overall, this patient exhibited a Grade VI cherubism according to the Motamedi and Raposo-Amaral grading systems [13, 14]. Because of the aesthetic and functional impairment (nasal obstruction, proptosis), partial surgical resection of the granuloma was performed without complication.

#### Genetic testing: sequencing analysis

Genetic counselling and testing were carried out. Direct sequencing of the *SH3BP2* gene identified a heterozygous transition mutation from G to A (c.1244G > A) in exon 9. This variation is expected to affect the amino acid sequence at the protein level, resulting in the substitution of Arg415 by Gln (p.Arg415Gln) (Fig. 1c). This variation has already been reported to be responsible for cherubism in other patients [1].

#### Pathologic and immunohistochemical examination

Histological analysis carried out on the granuloma showed moderate to dense cellular areas composed of numerous intermixed oval to spindle-shaped fibroblasts and osteoclast-like giant multinucleated cells. Some areas were predominantly composed of fibroblasts and had few giant multinucleated cells, occasionally situated within a more abundant collagenous stroma. Numerous vessels were observed, sometimes surrounded by perivascular hyalinosis (Fig. 1d). As previously described [15], the giant multinucleated cells were CD68 positive (Fig. 1e). The giant multinucleated cells presented in cherubism granulomas are usually TRAP positive as in this granuloma (Fig. 1f-g).

The severity of the cherubism was evaluated as previously described [15], by a nuclear and cytoplasmic NFATc1 immunostaining (Fig. 1h-i). These results are in accordance with our previous study [16].

Taken together, these data established the aggressive cherubism diagnosis.

### Explorations of systemic manifestations

#### Biological investigations

##### Standard biological examination

Blood results showed hypochromic anemia with thrombocytosis without sickle-cell anemia and with a normal hemoglobin electrophoresis. Serum electrolytes showed mild hyperphosphatemia but a normal serum calcium level (ionized as well as total). Plasma creatinine and urine calcium were normal. All of these data were adjusted for age (for details see Table 1). Prothrombin time and Kaolin Cephalin time were within the normal range, excluding hepatitis insufficiency.

**Table 1:** Serum levels of blood markers (pre-operative blood test) –elevated values are shown in red

***Mineral metabolism and bone turnover markers***		
**Serum marker**	**Normal range**	**Patient’s serum level**
Calcium (mmol/L)	2.20–2.70 (all ages)	2.37
Phosphate (mmol/L)	1.3–1.85 (all ages)	2.10
PTH (ng/mL)	10–50 (all ages) [17]	54
CTx (pmol/L)	3875–14,260 (6 years) [18]	18,678
Osteocalcin (μg/L)	45.8–128 (6 years) [18]	121
PINP (ng/mL)	324–895.2 (6 years) [18]	1480
25OHD (ng/mL)	30–60 (all ages)	34
1.25(OH)_2_D (pg/mL)	45–102 (prepubertal children > 3 years) [19]	49
BALP (ng/mL)	39.6–98.9 (6 years) [18]	126.6
***Inflammation exploration***		
**Serum marker**	**Normal range**	**Patient’s serum level**
CRP (mg/L)	< 6.0	2.9
IL-1 beta (pg/mL)	0–15	75.2
IL-6 (pg/mL)	0–8.6	4.1
IL-10 (pg/mL)	0–112	27.6
TNF alpha (pg/mL)	0–20	38.8

*PTH* parathyroid hormone; *CTx* beta-C-terminal telopeptide; *PINP* procollagen type I N-terminal Propeptide; *25OHD* 25-hydroxyvitamin D; *1.25(OH)*_*2*_*D* 1,25-dihydroxyvitamin D; *BALP* bone isoenzyme alkaline phosphatase; *CRP* C Reactive Protein; *IL* Interleukin; *TNF* Tumor Necrosis Factor

##### Bone and mineral metabolism parameters

The bone resorption marker beta C-terminal telopeptide of type I collagen (CTx), the bone formation markers bone isoenzyme alkaline phosphatase (BALP), procollagen type I N-terminal propeptide (P1NP) and osteocalcin were all elevated. Parathyroid hormone was subnormal and 25-hydroxyvitamin D and 1,25-dihydroxyvitamin D serum levels were normal (Table 1).

##### Inflammation exploration

C-reactive protein, fibrinogen, interleukin-6 and -10 serum levels were normal. TNFα (Tumor Necrosis Factor) and interleukin-1 levels were elevated (Table 1).

##### Radiological examinations

An abdominal ultrasound-scan showed slightly increased liver and spleen volumes (+ 1 SD) that were homogeneous and painless, without lymphadenopathy.

**Fig. 2:**
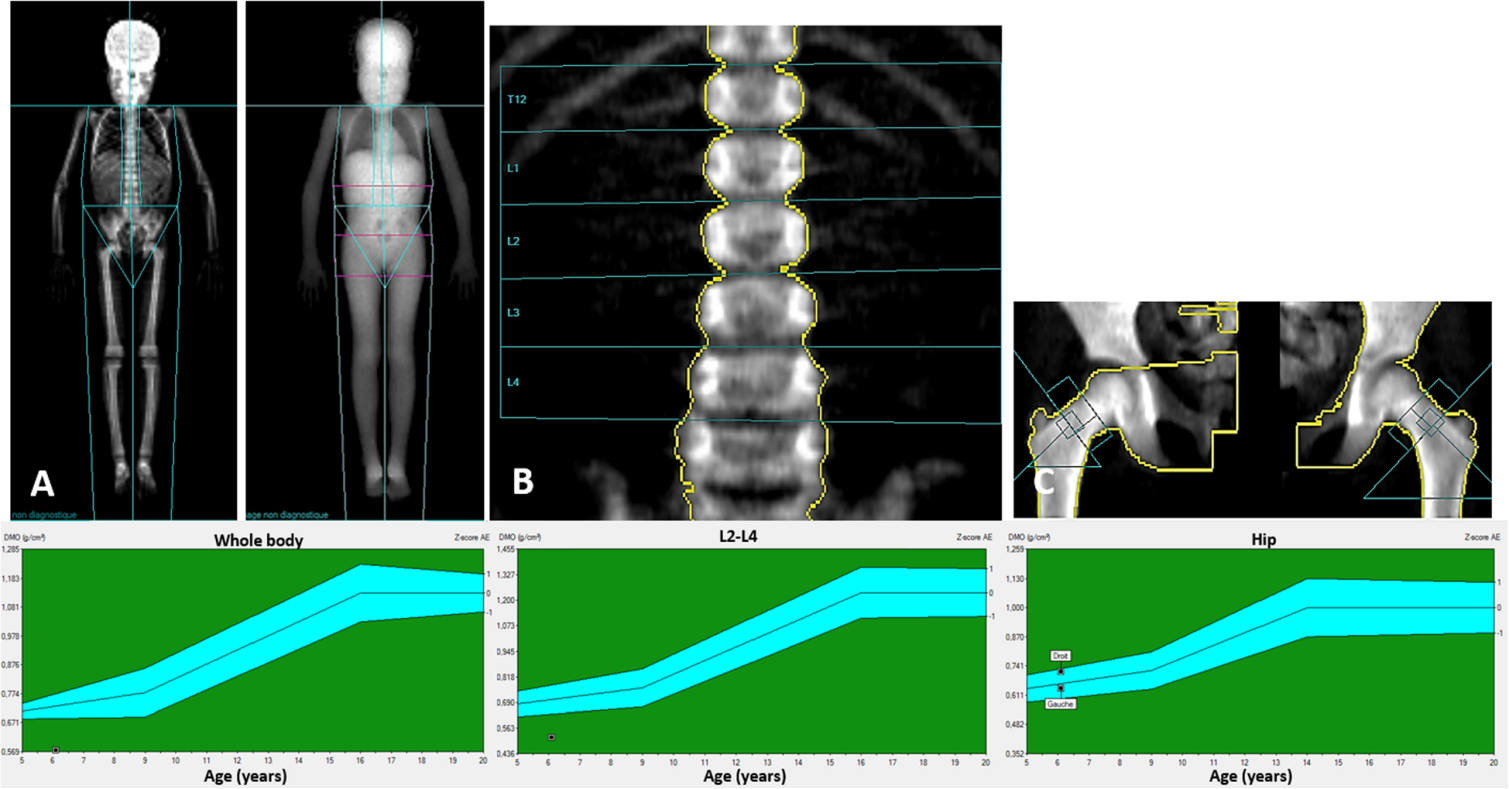
Presence of bone loss features revealed by areal Bone Mineral Density (aBMD) measurement for the whole body excluding the head (**a**), at the lumbar spine (**b**) and the hip (**c**). For each site, a representative DXA image and the Z-score are shown

Total body and lumbar spine (L2-L4) aBMD values were significantly low: *Z*-score = − 4.6 SD; − 2.8 SD, respectively. Total left hip aBMD Z-score was in the lower part of the normal range (Z-score = − 1.0 SD) (Fig. 2). aBMD Z-scores were then adjusted to age and ethnic group (GE Lunar Corp., Madison, WI, USA). The values of the aBMD Z-scores according to the type of adjustment are presented in Table 2.

**Table 2:** Dual X-ray absorptiometry: results of Z-scores according to the type of adjustment

	Unadjusted	Lunar, female pediatric reference curve adjusted for black ethnic origin	Lunar, female paediatric Caucasian reference curve	
	aBMD Z-score	aBMD Z-score for chronological age	aBMD Z-score for chronological age	aBMD Z-score for height age
Whole body	**- 4.6 SD**	**- 3.2 SD**	**- 2.2 SD**	**- 2.9 SD**
Lumbar spine (L2-L4)	**- 2.8 SD**	**- 2.6 SD**	**- 2.1 SD**	**- 2.0 SD**
Total left Hip	**- 1.0 SD**	**−0.3 SD**	**−0.3 SD**	**0.0 SD**

*aBMD* areal Bone Mineral Density

### Infectious disease exclusion

Viral serologic testing (hepatitis A, B, C; human immunodeficiency virus Epstein-Barr, Cytomegalovirus) was negative. Bacteriological and parasitological stool examinations were normal. Malaria microscopic diagnosis by staining thin and thick peripheral blood smears was negative.

### Search for a second-site bone mass-changing mutation

To rule out the presence of a mutation in a gene other than *SH3BP2* as being the cause of the low bone density observed in the patient, a panel of genes known to be associated with pathological bone mass changes was sequenced. The results of the gene panel sequencing did not reveal any pathological variants.

## Discussion and conclusions

About 400 cases of cherubism have been reported up to date in various ethnic groups worldwide. Severity and clinical expressivity of the disease range from no clinically detectable features to grotesquely deforming jaws leading to life threating conditions [3, 20, 21], as in the present case. This case study reports for the first time a cherubism patient with systemic manifestations, and hence questions the exclusive maxillofacial tropism of this pathology as described to date [22].

Recent genetic, molecular and cellular studies have provided critical insights into the pathogenic mechanisms of cherubism thanks in large part to the creation of knock-in mouse models expressing SH3BP2 mutations responsible for the human pathology [7, 23]. However, unlike human cherubism patient who are heterozygous for the SH3BP2 mutations, heterozygous mice do not exhibit any cherubism phenotype. In contrast, homozygous mutant mice develop severe systemic bone loss related to osteoclast hyperactivity as well as systemic inflammation due to hyperactive macrophages secreting elevated levels of TNF-α [7]. Despite this new knowledge, no investigations have yet explored systemic inflammatory and bone phenotypes in human patients, presuming the disease to be exclusively maxillofacial.

The diagnosis of aggressive cherubism in our patient was made according to the standard genetic, clinical and morphopathological analyses (Fig. 1). The patient exhibits an extremely severe case of cherubism with strong nuclear NFATc1 staining as previously described [16].

Furthermore, in this case report, we showed that the patient exhibits a systemic bone loss in addition to the maxillofacial involvement. The patient presented low bone density on DXA examination (whole body aBMD Z-score − 2.8 SD) and high bone turnover, as evidenced by elevated levels of CTx, a marker of bone resorption, and P1NP and osteocalcin, markers of bone formation [24]. We eliminated other potential aetiologies by blood tests and genetic studies (hemopathy, other chronic inflammatory diseases, immune deficiencies, gene panel sequencing and nutritional deficiency). Osteoporosis in chronic inflammatory disease has been described previously, especially in paediatric inflammatory bowel disease or chronic inflammatory conditions [25]. The principal involved mechanism is a chronic systemic inflammatory process, which activates osteoclastogenesis via circulating cytokines [26]. In the cherubism mouse model, bone loss is thought to be due to both chronic inflammation and direct stimulation of osteoclastogenic differentiation [7]. The involved mechanisms might be the same as those in the pathogenesis of human cherubism.

The patient’s low bone mass was characterized by whole body and lumbar spine aBMD Z-scores (Table 2). However, the total left hip aBMD Z-score did not show low bone mass. It has already been reported that measurement at the hip is not reliable in growing children [27]. Of note, the patient is Gabonese and her Z-scores were adjusted according to stature-for-age and sex. Concerning ethnic considerations, as there are no data for African populations, we used the LUNAR ethnicity adjustment for the black ethnic group. Thus, we cannot exclude a possible bias in the interpretation of the aBMD Z-score in this patient.

Simultaneously, the patient presented a homogeneous, painless hepatomegaly without hepatitits insufficiency, associated with splenomegaly. Because of her ethnic origin, parasitological, virology, and hematological examinations were performed to exclude tropical causes of hepatomegaly and splenomegaly.

Moreover, we detected an increase of interleukin-1 and TNF-α levels in the patient’s serum.

TNF-α expression has been shown in jaw lesions in human cherubism, but TNF-α serum level has not been reported [28]. In human cherubism, it remains unclear whether increased TNF-α production is primarily induced locally in jawbone lesions by macrophages, or by circulating myeloid cells. In our case, jawbone lesions began at the age of 3, and became rapidly more aggressive at the age of 6, with systemic bone loss and inflammation. This timeline highlights that jawbone lesions may have preceded systemic manifestations. However, TNF-α alone is not sufficient to induce osteoclast differentiation and activity, but synergistically potentiates with other cytokines, such as RANKL, IL-1 and TGF-β [29].

Most of the *SH3BP2* mutations causing cherubism are in the exon 9 of the gene, in a region coding for a 6 amino acid region (RSPPDG) between the PH and SH2 domains of the protein. Levaot et al. in 2011 [30] identified the Tankyrases (TNKS) as the proteins binding to this sequence. In addition, they demonstrated that TNKS by ribosylating Sh3bp2 allows its interaction with RNF146. The latter is then responsible for the ubiquitylation of Sh3bp2 hence its degradation [30]. Using the cherubism mouse model, Levaot et al. demonstrated that the Sh3bp2 mutation increased the stability of the protein, leading to its cytoplasmic accumulation [31]. Then, the increased Sh3bp2 quantity is able to enhance osteoclastogenesis and induce systemic bone loss as in our patient. However, it is not clear how this Sh3bp2 cytoplasmic accumulation in the osteoclasts primarily triggers a jaw osteolysis and only arising in deciduous or mixed dentition.

Myeloid Differentiation Factor 88 (MYD) is an adaptator protein that binds Toll-Like Receptor (TLR) and IL1R, to activate downstream transcription factors leading to cytokine production. TLR recognize pathogen-molecular patterns (PAMPs) from microorganism and damage associated molecular patterns (DAMPs) released by stressed cells or damaged tissues. In Sh3bp2^ki/ki^ mice, Yoshitaka et al. demonstrated that TLR2–4-MYD88 mediated pathway in hematopoietic cells was critical for cherubism phenotype development and that IL1R played a minor role in cherubism inflammation [32]. They showed that stimulation of TLR by PAMPs induced an Y183 Sh3bp2 phosphorylation by SYK, leading to an activation of NFκB pathway and a production of TNF-α in mutant macrophages and that SYK was primordial in the cherubism pathogenesis [32]. By creating germ-free Sh3bp2^ki/ki^ mice, they showed that bacterial stimulation was not the only trigger for cherubism and that DAMPs participated only partially to the activation of cherubism [32]. From this study, the authors proposed an explanation to the craniofacial localization of the human cherubism phenotype and the natural course of the disease. They suggested that human cherubism (as heterozygous mice) is a latent disease, activated by the detection of PAMPs from the microbial flora in the oral cavity or DAMPs secreted during teeth eruption or mechanical stress of mastication. This theory would explain the craniofacial localization of the cherubism in human, and more specifically, the DAMPs involvement during teeth eruption theory might explain the age of expression of the disease. Indeed, upon microbial stimuli on cherubism affected patient, TLR activation could initiate the process responsible for cherubism, and in severe cases, lead to systemic manifestations, by a vicious circle, as observed in autoinflammatory diseases.

In a previous study, we showed that multinucleated giant cells from granuloma are CD68-positive cells and hypothesized that these cells may differentiate into macrophages in non-aggressive cherubism and into osteoclasts in aggressive cherubism, stimulated by the NFATc1 pathway [15]. This same imbalance might be involved in the osteoporotic phenotype observed here. However more thorough explorations should be conducted before any definitive conclusions are drawn. The human cherubism trigger has yet to be identified as our previous study suggests a more secondary role for TNF-α in the human pathogenesis [15], whereas TNF-α had been largely incriminated in the mouse cherubism phenotype [33].

We effectively ruled out mutations other than that in the *SH3BP2* gene as causing the low bone mass phenotype of our patient by sequencing 50 genes known or suggested to cause alterations in bone mineral density and not finding any pathological variants. Obviously, our a priori analysis cannot totally rule out the existence of a causative second-site mutation in a gene not included in our panel; nonetheless, our results strongly suggest that the systemic bone phenotype of our patient is due to the SH3BP2 mutation.

We may hypothesize from this case that cherubism is a systemic bone disease with a systemic skeletal phenotype that could follow the evolutionary time course of the jaw lesions. To confirm this hypothesis, a study of the osseous and inflammatory phenotypes in a larger series of cherubism patients should be carried out. We suggest also paying more attention to a potential cherubism systemic skeletal phenotype during patient management. If this systemic skeletal cherubism phenotype is confirmed, it would simplify treatment of severe cherubism patients and allay reservations about applying a systemic treatment such as those recently published (tacrolimus [34] or imatinib [35]), to a previously believed jaw-localised disease.

## Data Availability

All the data supporting our findings are contained within the manuscript.

## Abbreviations

aBMD: areal bone mineral density (aBMD)
NFATc1: Nuclear factor of activated T-cells, cytoplasmic 1
